# mxnorm: An R Package to Normalize Multiplexed Imaging Data

**DOI:** 10.21105/joss.04180

**Published:** 2022-03-30

**Authors:** Coleman Harris, Julia Wrobel, Simon Vandekar

**Affiliations:** 1 Department of Biostatistics, Vanderbilt University Medical Center, Nashville, TN, USA; 2 Department of Biostatistics & Informatics, Colorado School of Public Health, Aurora, CO, USA

## Abstract

Multiplexed imaging is an emerging single-cell assay that can be used to understand and analyze complex processes in tissue-based cancers, autoimmune disorders, and more. These imaging technologies, which include co-detection by indexing (CODEX), multiplexed ion beam imaging (MIBI), and multiplexed immunofluorescence imaging (MxIF), provide detailed information about spatial interactions between cells (Angelo et al., 2014; Gerdes et al., 2013; Goltsev et al., 2018). Multiplexed imaging experiments generate data across hundreds of slides and images, often resulting in terabytes of complex data to analyze through imaging analysis pipelines. Methods are rapidly developing to improve particular parts of the pipeline, including software packages in R and Python like spatialTime, imcRtools, MCMICR0, and Squidpy (Creed et al., 2021; Palla et al., 2021; Schapiro et al., 2021; Windhager et al., 2021). An important, but understudied component of this pipeline is the analysis of technical variation within this complex data source – intensity normalization is one way to remove this technical variability. The combination of disparate pre-processing pipelines, imaging variables, optical effects, and within-slide dependencies create batch and slide effects that can be reduced via normalization methods. Current state-of-the-art methods vary heavily across research labs and image acquisition platforms, without one singular method that is uniformly robust – optimal statistical methods seek to improve similarity across images and slides by removing this technical variability while maintaining the underlying biological signal in the data.

mxnorm is open-source software built with R and S3 methods that implements, evaluates, and visualizes normalization techniques for multiplexed imaging data. Extending methodology described in Harris et al. (2022), we intend to set a foundation for the evaluation of multiplexed imaging normalization methods in R. This easily allows users to extend normalization methods into the field, and provides a robust evaluation framework to measure both technical variability and the efficacy of various normalization methods. One key component of the R package is the ability to supply user-defined normalization methods and thresholding algorithms to assess normalization in multiplexed imaging data. Core features, usage details, and extensive tutorials are available in the package documentation and vignette on CRAN and the software repository.

## Statement of need

Multiplexed imaging measures intensities of dozens of antibody and protein markers at the single-cell level while preserving cell spatial coordinates. This allows single-cell analyses to be performed on biological samples like tissues and tumors, much like single-cell RNA sequencing, with the added benefit of *in situ* coordinates to better capture spatial interactions between individual cells (Chen et al., 2021; McKinley et al., 2022). Current research using platforms like MxIF and MIBI demonstrate this growing field that seeks to better understand cell-cell populations in cancer, pre-cancer, and various biological research contexts (Gerdes et al., 2013; Ptacek et al., 2020).

In contrast to the field of sequencing & micro-array data and the established software, analysis, and methods therein, multiplexed imaging lacks established analysis standards, pipelines, and methods. Recent developments in multiplexed imaging seek to address the broad lack of standardized tools – the MCMICRO pipeline seeks to provide a set of open-source, reproducible analyses to transform whole-slide images into single-cell data (Schapiro et al., 2021). Researchers in the field have also developed a ground truth dataset to evaluate differences in batch effects and normalization methods (Graf et al., 2022), while other open issues in the field that may produce open-source solutions include tissue segmentation, end-to-end image processing, and removal of image artifacts. With this diversity of open issues in multiplexed imaging, our work focuses specifically on normalization methods and evaluating these results in multiplexed imaging data. Namely, standard normalization software in the sequencing field includes open-source packages in R and Python like sva, limma, and Scanorama (Hie et al., 2019; Leek et al., 2012; Smyth, 2005), but an analogue for evaluating and developing normalization methods does not exist for multiplexed imaging data.

We recently proposed and evaluated several normalization methods for multiplexed imaging data, which along with other recent work shows that normalization methods are important in reducing slide-to-slide variation (Burlingame et al., 2021; Chang et al., 2020; Harris et al., 2022). These recently developed algorithms are the beginning of contributions to normalization literature, but lack a simple, user-friendly implementation. Further, there is no software researchers can use to develop and evaluate normalization methods in their own multiplexed imaging data; multiplexed imaging software is limited mostly to Matlab, Python, and only a scattered few R packages exist. Two prominent packages, cytomapper and giotto, contain open-source implementations for analysis and visualization of highly multiplexed images (Dries et al., 2021; Eling et al., 2020), but do not explicitly address normalization of the single-cell intensity data. Hence, there is a major lack of available tools for researchers to explore, evaluate, and analyze normalization methods in multiplexed imaging data. The mxnorm package provides this framework, with easy-to-implement and customizable normalization methods along with a foundation for evaluating their utility in the multiplexed imaging field.

## Functionality

**Figure 1:**
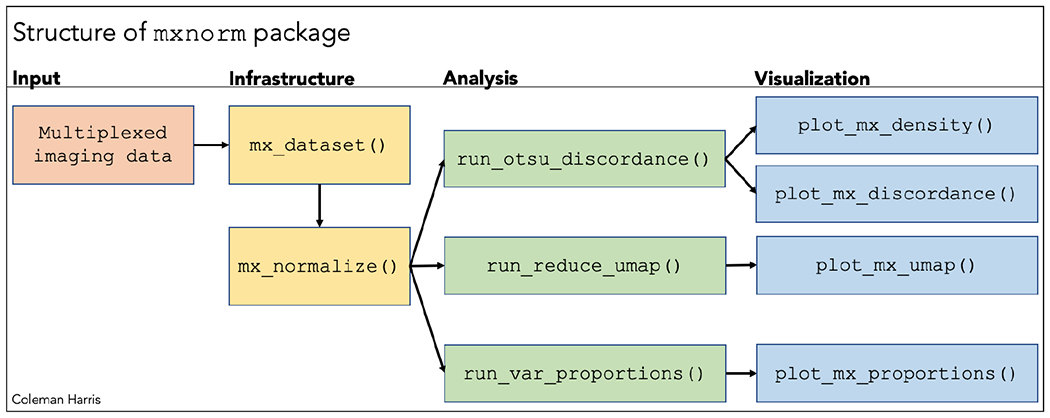
Basic structure of the mxnorm package and associated functions

As shown in Figure 1, there are three main types of functions implemented in the mxnorm package – infrastructure, analysis, and visualization. The first infrastructure function, mx_dataset( ), specifies and creates the S3 object used throughout the analysis, while the mx_normalize( ) function provides a routine to normalize the multiplexed imaging data, which specifically allows for normalization algorithms defined by the user. Each of the three analysis functions provides methods to run specific analyses that test for slide-to-slide variation and preservation of biological signal for the normalized and unnormalized data, while the four visualization functions provide methods to generate ggplot2 plots to assess the results. We also extend the summary( ) generic function to the mx_dataset S3 object to provide further statistics and summaries.

The statistical methodology underlying the methods we implemented in mxnorm builds upon existing work in both R and Python. Normalization algorithms available in mx_normalize( ) leverage methodology derived in the ComBat paper, the fda package, and the tidyverse framework (Johnson et al., 2007; Ramsay et al., 2021; Wickham et al., 2019). The threshold discordance methods available in run_otsu_discordance( ) leverage methodology from Otsu’s original paper and the scikit-image implementation of Otsu thresholding in Python (Otsu, 1979; Walt et al., 2014). Our implementation of the UMAP algorithm in run_reduce_umap( ) leverages both the UMAP paper and the uwot implementation of the UMAP algorithm in R (McInnes et al., 2018; Melville, 2021). The random effects modeling options available in run_var_proportions( ) leverage the lme4 R package (Bates et al., 2015). Even more information for the statistical methodology behind these normalization and analysis methods are detailed further in our package vignette and in the methods paper (Harris et al., 2022).

## A minimal example

The following code is a simplified example of a normalization analysis applied to the sample dataset included in the mxnorm package, mx_sample. Here we specify the creation of the S3 object, normalize using the mean_divide method, run a set of analyses to compare our normalized data with the unnormalized data, and finally generate summary statistics and plots to understand the results.

## load package

library(mxnorm)

## create S3 object & normalize

mx_data = mx_dataset(mx_sample, ”slide_id”, ”image_id”,

             c(”marker1_vals”,”marker2_vals”,”marker3_vals”),

             c(”metadata1_vals”))

mx_data = mx_normalize(mx_data, ”mean_divide”, ”None”)

## run analyses

mx_data = run_otsu_discordance(mx_data, ”both”)

mx_data = run_reduce_umap(mx_data, ”both”,

                c(”marker1_vals”,”marker2_vals”,”marker3_vals”))

mx_data = run_var_proportions(mx_data, ”both”)

## results and plots

summ_mx_data = summary(mx_data)

p1 = plot_mx_denstiy(mx_data)

p2 = plot_mx_discordance(mx_data)

p3 = plot_mx_umap(mx_data, ”slide_id”)

p4 = plot_mx_proportions(mx_data)
